# Supplementary material for: Combination of Meropenem and Zinc Oxide Nanoparticles; Antimicrobial Synergism, Exaggerated Antibiofilm Activity, and Efficient Therapeutic Strategy against Bacterial Keratitis
Source: Antibiotics (Basel). 2022 Oct 7;11(10):1374. doi: 10.3390/antibiotics11101374 (PMC9598448; doi:10.3390/antibiotics11101374)
Supplement: Supplementary file 1 [file antibiotics-11-01374-s001.zip › antibiotics-1956497-supplementary.pdf]

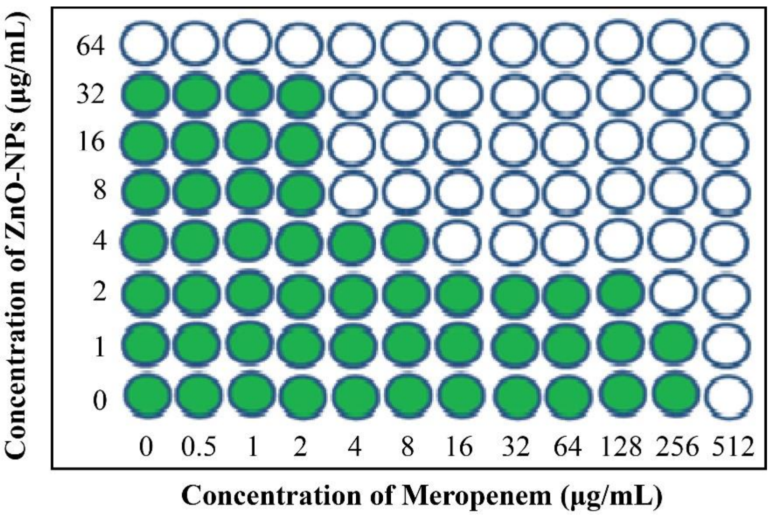

Figure S1. A schematic diagram of the distribution of meropenem and ZnO-NPs concentration in a 96-well plate

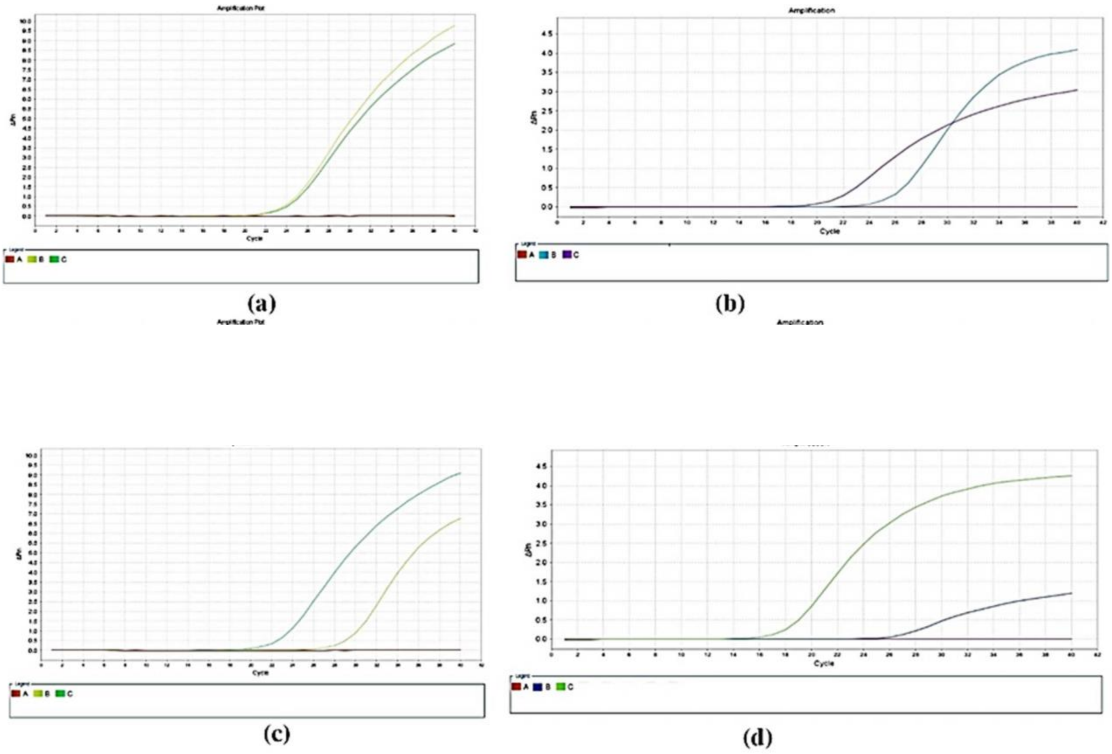

Figure S2. Data sheet of RT-PCR showing amplification curves of (a) 16s rRNA structural genes and biofilm genes (b) fliC, (c) lasR and (d) pslA of carbapenem-resistant *P. aeruginosa* PU15.
